# Associations of Smoking and Drinking with New Lipid-Related Indices in Women with Hyperglycemia

**DOI:** 10.1089/whr.2020.0100

**Published:** 2021-01-29

**Authors:** Ichiro Wakabayashi

**Affiliations:** Department of Environmental and Preventive Medicine, Hyogo College of Medicine, Nishinomiya, Japan.

**Keywords:** alcohol, blood lipids, cardiovascular disease, diabetes, hyperglycemia, smoking

## Abstract

***Background:*** Lipid-related indices are useful for early detection of the risk of cardiovascular disease. The relationships of smoking and alcohol drinking with lipid-related indices in women with diabetes remain to be clarified.

***Methods:*** In female participants with hyperglycemia, four lipid-related indices, ratio of low-density lipoprotein cholesterol to high-density lipoprotein cholesterol (LDL-C/HDL-C ratio), atherogenic index of plasma (AIP), lipid accumulation product (LAP), and cardiometabolic index (CMI), were compared in smokers and nonsmokers and in occasional drinkers, regular drinkers, and nondrinkers. Analysis of covariance and logistic regression analysis were used for comparison with adjustment for age, hemoglobin A1c, history of regular exercise, and history of alcohol drinking or smoking.

***Results:*** Mean levels of LDL-C/HDL-C ratio, AIP, and CMI were significantly higher in smokers than in nonsmokers, and the odds ratios in smokers versus nonsmokers for high LDL-C/HDL-C ratio, high AIP, and high CMI were significantly higher than the reference level. These differences in mean levels and odds ratios were not found in analysis of LAP. Mean levels of LDL-C/HDL-C ratio, LAP, AIP, and CMI were significantly lower in regular drinkers than in nondrinkers. The odds ratios versus nondrinkers for high LDL-C/HDL-C ratio, high AIP, high LAP, and high CMI in regular drinkers were significantly lower than the reference level. The odds ratios versus nondrinkers for high LDL-C/HDL-C ratio, high LAP, and high CMI in occasional drinkers were also significantly lower than the reference level.

***Conclusions:*** In women with hyperglycemia, smoking was positively associated with LDL-C/HDL-C ratio, AIP, and CMI, and habitual alcohol drinking was inversely associated with LDL-C/HDL-C ratio, AIP, LAP, and CMI. Thus, LDL-C/HDL-C ratio, AIP, and CMI are thought to be affected by both smoking and alcohol drinking, which accelerates and suppresses atherosclerotic progression, respectively.

## Introduction

Cardiovascular complications are major factors determining the prognosis of patients with diabetes, and early detection of atherosclerosis and reducing cardiovascular risk are important for prevention of cardiovascular events, including ischemic heart disease, stroke, and peripheral arterial disease.^1^ Dyslipidemia is a major risk factor for atherosclerotic disease,^2,3^ and patients with diabetes are more prone to have dyslipidemia than those without diabetes.^4^ Lipid-related index, which is calculated by using lipids alone or lipids and adiposity index, is useful for evaluating the cardiovascular risk related to dyslipidemia. The ratio of low-density lipoprotein cholesterol (LDL-C) to high-density lipoprotein cholesterol (HDL-C) is a classical atherogenic index and a predictor of ischemic heart disease.^5^ Several lipid-related indices, including atherogenic index of plasma (AIP), lipid accumulation product (LAP), and cardiometabolic index (CMI) have recently been proposed. AIP is defined as the logarithm of the ratio of triglycerides (TGs) to HDL-C^6^ and it has been reported to be a strong predictor of ischemic heart disease.^7,8^ LAP is calculated as the product of triglycerides and modified waist circumference (WC)^9^ and it has been shown to be associated with the risks of diabetes and cardiovascular disease.^10–12^ CMI is defined as the product of waist-to-height ratio and triglycerides-to-HDL-C ratio^13^ and it has been shown to be associated with the degree of atherosclerosis in patients with peripheral arterial disease and with arterial stiffness in a general population.^14,15^ Thus, LAP and CMI are indices reflecting both adiposity and blood lipids. We sometimes experience diverse results of examinations of adiposity and blood lipids, for example, high triglycerides and large WC but high HDL-C. Thus, in such cases, overall assessment of the lipid profile and adiposity is needed when a risk for future cardiovascular disease is speculated after health checkup examinations. Interestingly, in a previous prospective study, TG/HDL-C ratio and total cholesterol/HDL-C ratio were shown to be better predictors of coronary arterial disease than lipid (HDL-C or LDL-C) alone.^16^ Therefore, lipid-related indices are thought to be practically useful for clinicians to access cardiovascular risk.

Cigarette smoking and alcohol drinking are representative lifestyles influencing incidental cardiovascular diseases. Cigarette smoking is a major cardiovascular risk factor and deteriorates the blood lipid profile: HDL-C and triglycerides are lower and higher, respectively, in smokers than in nonsmokers.^17^ Habitual drinking has diverse effects on the levels of blood lipids: HDL-C and triglycerides are higher in drinkers than in nondrinkers, and LDL-C is lower in drinkers than in nondrinkers.^18^ Adiposity has also been shown to be influenced by smoking^19,20^ and alcohol drinking.^21,22^ Thus, lipid-related indices are influenced by smoking and drinking through their multiple effects on blood lipid profiles and adiposity. In our previous studies using a database of a general population, lipid-related indices were found to be higher in smokers than in nonsmokers,^23,24^ whereas light-to-moderate drinkers showed lower lipid-related indices than those in nondrinkers.^25–27^ Moreover, CMI has been shown to be lower in light-to-moderate drinkers than in nondrinkers in men with diabetes.^28^ Women with diabetes have been shown to have a more adverse cardiometabolic profile, including visceral obesity, high pulse pressure, high LDL-C, low HDL-C, and metabolic syndrome, than that in men.^29^ However, there have been few studies on the relationships between lifestyles and recently proposed lipid-related indices in women with glucose intolerance.

Patients with diabetes are known to be prone to have dyslipidemia and obesity, and a cardiovascular complication is an important determinant for their prognosis. Therefore, in addition to better control of blood glucose level, earlier correction of dyslipidemia is crucial for prevention of cardiovascular disease in diabetes patients, and lipid-related indices are thus thought to be useful biomarkers for overall assessment of cardiovascular risk related to a disorder of blood lipids. Generally, the percentage of smokers is lower and the amount of cigarette consumption is smaller in women in Asian countries than in women in Western countries (recent percentages of smokers in women in the general population: 7.2% in Japan, 5.2% in Korea, and 2.7% in China vs. 18.7% in the United States, 18.6% in Germany, and 28.7% in France),^30^ and thus relationships of smoking with blood lipids are speculated to be generally weaker in Asian women. However, there is limited information on the relationships between smoking and lipid-related indices in Asian women. Smokers are prone to be lean compared with nonsmokers, while smoking is a risk factor for dyslipidemia. Thus, it would be interesting to determine how lipid-related indices reflecting both blood lipids and adiposity are influenced by smoking in Asian women, although both smoking and dyslipidemia are confirmed independent cardiovascular risk factors.

The purpose of this study was therefore to elucidate the relationships of habitual smoking and alcohol drinking with lipid-related indices including LDL-C/HDL-C ratio, AIP, LAP and CMI in Japanese women with hyperglycemia. Since the proportions of heavy smokers and drinkers are very small in women, lipid-related indices in women with hyperglycemia were simply compared in smokers and nonsmokers and in groups of different frequencies of alcohol drinking (non-, occasional, and regular drinkers).

## Methods

### Participants

The participants in the original database were Japanese female workers ages 35–70 years (*n* = 18,793) who had received periodic health checkups at workplaces in Yamagata Prefecture in Japan. This study was approved by the Hyogo College of Medicine Ethics Committee (No. 3003 in 2018). Histories of alcohol consumption, cigarette smoking, illness, and therapy for illness were surveyed by questionnaires. In the questionnaires, participants were required to identify any conditions for which they were receiving treatment. Those who had been receiving medication therapy for dyslipidemia were excluded from the participants of this study. Histories of cigarette smoking, alcohol consumption, regular exercise (almost every day with exercise for 30 minutes or longer per day), and illness were also surveyed by the questionnaires. According to the criteria of hyperglycemia by hemoglobin A1c level and a history of medication therapy for diabetes as mentioned below, participants with hyperglycemia (*n* = 2,137) were extracted as the subjects of this study from the original database. A history of diabetes was surveyed by a questionnaire using the simple question “Do you have a history of receiving medication therapy for diabetes?.” Since it is not always easy for people to remember drug names correctly in a health checkup, specific content of medication was not asked in the questionnaire.

In the self-written questionnaire paper, participants were first asked “Are you a habitual cigarette smoker?” Cigarette smokers were defined as participants who had smoked for 6 months or longer and had smoked for the past month or longer. Then the participants who had been smokers were further asked “What is your average cigarette consumption per day?.” The response categories for this question were “less than 21 cigarettes per day,” “21 or more and less than 41 cigarettes per day” and “41 or more cigarettes per day.” In this study, only two categories of smoking, nonsmokers and smokers, were used for analysis because the percentage of heavy smokers who smoked 21 or more cigarettes per day was very low, 0.60% (*n* = 13), in overall participants.

Average alcohol consumption of each participant per week was reported in the questionnaires. Frequency of habitual alcohol drinking was asked in the questionnaires as “How frequently do you drink alcohol?.” Frequency of weekly alcohol drinking was categorized as “every day” (regular drinkers), “sometimes” (occasional drinkers), and “never” (nondrinkers).

### Measurements

Height and body weight were measured with the subjects wearing light clothes at the health checkup. Body mass index (BMI) was calculated as weight in kilograms divided by the square of height in meters. WC was measured at the navel level according to the recommendation of the definition of the Japanese Committee for the Diagnostic Criteria of Metabolic Syndrome,^31^ and visceral obesity was evaluated by the ratio of WC (cm) to height (cm) (waist-to-height ratio).

Fasted blood was sampled from each participant in the morning, and serum triglyceride, HDL-C, and LDL-C levels were measured by enzymatic methods using commercial kits, pureauto S TG-N, cholestest N-HDL, and cholestest LDL (Sekisui Medical Co., Ltd, Tokyo, Japan), respectively. Hemoglobin A1c was measured by the NGSP (National Glycohemoglobin Standardization Program)-approved technique using the latex cohesion method with a commercial kit (Determiner HbA1c; Kyowa Medex, Tokyo, Japan). Since the standards of hemoglobin A1c used for measurement are different in the NGSP method and JDS (the Japan Diabetes Society) method, hemoglobin A1c values were calibrated by using a formula proposed by the JDS^32^: hemoglobin A1c (NGSP) (%) = 1.02 × hemoglobin A1c (JDS) (%) + 0.25%. Subjects with hyperglycemia were defined as those showing hemoglobin A1c levels of 5.7% or higher and/or those receiving medication therapy for diabetes. The coefficients of variation for reproducibility of measurement were ≤3% for triglycerides, ≤5% for HDL-C, ≤5% for LDL-C, and ≤5% for hemoglobin A1c. The cutoff value used for high LDL-C/HDL-C was 2.80, which was determined by using the cutoff values of high LDL-C (140 mg/dL) and low HDL-C (50 mg/dL) for women.^33,34^ Since a cutoff value for high AIP has not been established, high AIP values were defined as those of the participants in the highest quartile group for AIP in the general population in the original database, in which the cutoff value of AIP for the highest quartile was −0.0649. LAP was determined by using TG level and WC as follows: LAP = TG (mmol/L) × [WC (cm) −58].^9^ The cutoff value for high LAP was defined as 21.1.^35^ CMI was calculated as the product of waist-to-height ratio and TG/HDL-C (mg/dL/mg/dL), and the cutoff value used for high CMI was 0.800.^13^

### Statistical analysis

For continuous variables, means of each variable were compared between nonsmokers and smokers by using Student's *t*-test and were compared between non, occasional, and regular drinkers by using analysis of variance (ANOVA) followed by Scheffé's F-test as a *post hoc* test in univariate analysis. In multivariate analysis, the mean levels of each variable were compared by using analysis of covariance (ANCOVA) followed by Student's *t*-test after Bonferroni correction. Since triglycerides, LAP, and CMI did not show normal distributions, they were used after logarithmic transformation in ANCOVA. Participants showing WC of 58 cm or smaller (*n* = 5) were excluded from subjects in ANCOVA for LAP because their values for log-transformation were zero or under zero and thus log-transformed LAP could not be calculated. In logistic regression analysis, odds ratios of smokers versus nonsmokers and odds ratios of occasional and regular drinkers versus nondrinkers for each variable (high LDL-C/HDL-C, high AIP, high LAP, or high CMI) were estimated.

Age, hemoglobin A1c, and habit of regular exercise were adjusted in ANCOVA and multivariate logistic regression analysis. A habit of alcohol drinking or smoking was also added to the covariates and explanatory variables in analyses for two smoker groups (nonsmokers and smokers) or three drinker groups (non, occasional, and regular drinkers). In addition, BMI was adjusted in analysis of variables, except for waist-to-height ratio, LAP, and CMI since these indices are calculated by using WC, which is strongly correlated with BMI, and both of them are adiposity-related indices.

All *p*-values are two-sided and values of *p* < 0.05 were considered to indicate statistical significance. Statistical analyses were performed using a computer software program (IBM SPSS Statistics for Windows, Version 25.0.; IBM Corp., Armonk, NY).

## Results

### Characteristics of the overall subjects

Table 1 shows characteristics of the overall subjects. The proportions of smokers, occasional drinkers, and regular drinkers were 13.1%, 23.5%, and 5.2%, respectively. Mean WC and waist-to-height ratio were 83.8 cm and 0.541, respectively. Mean hemoglobin A1c was 6.24% and the proportion of subjects with diabetes was 19.7%. About 10% of the subjects were receiving medication therapy for diabetes. About two-thirds of the subjects showed high LAP or high CMI levels.

**Table 1.** Characteristics of the Overall Subjects

Variable	Value in overall subjects
Number	2,137
Age, years	53.2 ± 7.0
Smokers, %	13.1
Drinkers, %	Occasional, 23.5; regular 5.2
Regular exercise, %	6.5
Height, cm	154.9 ± 5.7
Body weight, kg	59.0 ± 11.5
Waist circumference, cm	83.8 ± 10.6
BMI	24.6 ± 4.4
Waist-to-height ratio	0.541 ± 0.070
Hemoglobin A1c, %	6.24 ± 0.99
Therapy for diabetes, %	9.7
Diabetes mellitus, %	19.7
Triglycerides, mg/dL	111 (77, 162)
HDL-C, mg/dL	59.4 ± 14.7
LDL-C, mg/dL	129.7 ± 31.3
LDL-C/HDL-C ratio	2.33 ± 0.83
AIP	−0.072 ± 0.314
LAP	31.8 (18.0, 53.8)
CMI	1.05 (0.62, 1.78)
High LDL-C/HDL-C ratio, %	8.7
High AIP, %	49.3
High LAP, %	68.8
High CMI, %	63.9

Shown are number, frequency, mean with standard deviation, and median with 25 and 75 percentile values in parenthesis of each variable.

AIP, atherogenic index of plasma; BMI, body mass index; CMI, cardiometabolic index; HDL-C, high-density lipoprotein cholesterol; LAP, lipid accumulation product; LDL-C, low-density lipoprotein cholesterol.

### Comparison of each lipid-related index in smokers and nonsmokers

Mean or median levels of each lipid-related variable were compared in smokers and nonsmokers in univariate analysis (Table 2) and multivariate analysis (Table 3). In the multivariate analysis, values of LAP and CMI were used after logarithmic transformation. LDL-C/HDL-C ratio, AIP, and CMI were significantly higher in smokers than in nonsmokers, whereas LAP was not significantly different between smokers and nonsmokers.

**Table 2.** Comparison of Mean or Median Levels of Lipid-Related Indices Between Nonsmokers and Smokers in Women with Hyperglycemia in Univariate Analysis

Lipid-related index	Nonsmokers	Smokers
LDL-C/HDL-C	2.308 (2.271 to 2.345)	2.470 (2.362 to 2.578)^**^
AIP	−0.081 (−0.095 to −0.067)	−0.007 (−0.045 to 0.032)^**^
LAP	31.5 (18.0 to 53.0)	33.8 (17.9 to 61.1)
CMI	1.03 (0.61 to 1.76)	1.17 (0.70 to 2.00)^*^

Shown are mean levels with 95% confidence intervals in parentheses for LDL-C/HDL-C ratio and AIP and medians with 25 and 75 percentile values in parentheses for LAP and CMI in smokers and nonsmokers. Asterisks denote significant differences from nonsmokers (^*^p < 0.05; ^**^p < 0.01).

**Table 3.** Comparison of Mean Levels of Lipid-Related Indices Between Nonsmokers and Smokers in Women with Hyperglycemia in Multivariate Analysis

Lipid-related index	Nonsmokers	Smokers
LDL-C/HDL-C	2.295 (2.260 to 2.331)	2.537 (2.444 to 2.629)^**^
AIP	−0.084 (−0.097 to −0.071)	0.013 (−0.022 to 0.048)^**^
Log-LAP	1.467 (1.450 to 1.484)	1.480 (1.435 to 1.525)
Log-CMI	0.010 (−0.006 to 0.025)	0.078 (0.038 to 0.118)^**^

Mean levels with 95% confidence intervals in parentheses for each variable in smokers and nonsmokers are shown. Age, hemoglobin A1c, and histories of alcohol drinking and regular exercise were adjusted. In addition, BMI was adjusted in analysis of LDL-C/HDL-C ratio and AIP. Asterisks denote significant differences from nonsmokers (^**^p < 0.01).

### Odds ratios of smokers versus nonsmokers for high levels of each lipid-related index

Table 4 shows the results of logistic regression analysis for the relationship of smoking with high levels of each lipid-related index. Both in univariate and multivariate analyses, odds ratios for high LDL-C/HDL-C ratio, high AIP, and high CMI were significantly higher than the reference level. The odds ratio for high LAP was not significantly different from the reference level in both univariate and multivariate analyses.

**Table 4.** Relationships of Smoking with Abnormalities of Lipid-Related Indices in Women with Hyperglycemia

High lipid-related index	Nonsmokers	Smokers
High LDL-C/HDL-C (≥2.80)		
Crude	1.00	1.62 (1.10 to 2.40)^*^
Adjusted	1.00	1.82 (1.21 to 2.76)^**^
High AIP (≥ −0.0649)		
Crude	1.00	1.53 (1.18 to 1.97)^**^
Adjusted	1.00	1.84 (1.39 to 2.42)^**^
High LAP (≥21.1)		
Crude	1.00	1.00 (0.76 to 1.31)
Adjusted	1.00	1.04 (0.79 to 1.38)
High CMI (≥0.800)		
Crude	1.00	1.31 (1.00 to 1.72)^*^
Adjusted	1.00	1.42 (1.07 to 1.88)^*^

Odds ratios with 95% confidence intervals in parentheses for each variable in smokers versus nonsmokers are shown. In multivariate analysis, age, hemoglobin A1c, and histories of alcohol drinking and regular exercise were adjusted. In addition, BMI was adjusted in analysis of high LDL-C/HDL-C ratio and high AIP. Asterisks denote significant differences from the reference level of 1.00 (^*^p < 0.05; ^**^p < 0.01).

### Comparison of each component of the lipid-related indices in smokers and nonsmokers

Table 5 shows mean levels of the four components of the lipid-related indices in smokers and nonsmokers. Both in univariate and multivariate analyses, waist-to-height ratio and HDL-C was significantly lower and log-transformed triglycerides were significantly higher in smokers than in nonsmokers. LDL-C was significantly higher in smokers than in nonsmokers in multivariate analysis but not in univariate analysis.

**Table 5.** Comparison of Mean Levels of the Components of Lipid-Related Indices Between Nonsmokers and Smokers in Women with Hyperglycemia

Component of lipid-related index	Nonsmokers	Smokers
Waist-to-height ratio		
Univariate	0.543 (0.540 to 0.546)	0.529 (0.521 to 0.538)^**^
Multivariate	0.543 (0.540 to 0.546)	0.529 (0.521 to 0.538)^**^
Log-triglycerides, mg/dL		
Univariate	2.042 (2.031 to 2.053)	2.099 (2.069 to 2.130)^**^
Multivariate	2.040 (2.030 to 2.051)	2.110 (2.082 to 2.138)^**^
HDL-C, mg/dL		
Univariate	59.7 (59.0 to 60.4)	57.3 (55.7 to 59.0)^**^
Multivariate	59.9 (59.3 to 60.5)	56.3 (54.5 to 58.1)^**^
LDL-C, mg/dL		
Univariate	129.4 (127.9 to 130.8)	132.1 (128.2 to 136.0)
Multivariate	129.1 (127.7 to 130.5)	134.0 (130.4 to 137.7)^*^

Mean levels with 95% confidence intervals in parentheses for each variable in smokers and nonsmokers are shown. In multivariate analysis, age, hemoglobin A1c, and histories of alcohol drinking and regular exercise were adjusted. In addition, BMI was adjusted in analysis of variables, except for waist-to-height ratio. Asterisks denote significant differences from nonsmokers (^*^p < 0.05; ^**^p < 0.01).

### Comparison of each lipid-related index in drinkers and nondrinkers

Mean or median levels of each lipid-related variable were compared in non-, occasional, and regular drinkers in univariate analysis (Table 6) and multivariate analysis (Table 7). In the multivariate analysis, values of LAP and CMI were used after logarithmic transformation. LDL-C/HDL-C ratio, AIP, LAP, and CMI were significantly lower in regular drinkers than in nondrinkers. LDL-C/HDL-C ratio was also significantly lower in occasional drinkers than in nondrinkers.

**Table 6.** Comparison of Mean or Median Levels of Lipid-Related Indices Among Non-, Occasional, and Regular Drinkers in Women with Hyperglycemia in Univariate Analysis

Lipid-related index	Drinkers		
	Non	Occasional	Regular
LDL-C/HDL-C	2.382 (2.341 to 2.423)	2.264 (2.192 to 2.337)^*^	1.857 (1.711 to 2.003)^**^
AIP	−0.061 (−0.076 to −0.045)	−0.081 (−0.109 to −0.053)	−0.180 (−0.241 to −0.120)^**^
LAP	31.9 (18.6 to 53.6)	32.1 (17.9 to 56.2)	26.4 (12.2 to 42.0)^*^
CMI	1.08 (0.64 to 1.82)	1.01 (0.58 to 1.76)	0.76 (0.43 to 1.34)^**^

Shown are mean levels with 95% confidence intervals in parentheses for LDL-C/HDL-C ratio and AIP and medians with 25 and 75 percentile values in parentheses for LAP and CMI in non-, occasional, and regular drinkers. Asterisks denote significant differences from nondrinkers (^*^p < 0.05; ^**^p < 0.01).

**Table 7.** Comparison of Mean Levels of Lipid-Related Indices Among Non-, Occasional, and Regular Drinkers in Women with Hyperglycemia in Multivariate Analysis

Lipid-related index	Drinkers		
	Non	Occasional	Regular
LDL-C/HDL-C	2.382 (2.343 to 2.421)	2.249 (2.182 to 2.317)^**^	1.923 (1.778 to 2.067)^**^
AIP	−0.061 (−0.076 to −0.046)	−0.085 (−0.110 to −0.059)	−0.152 (−0.207 to −0.097)^**^
Log-LAP	1.478 (1.459 to 1.497)	1.463 (1.430 to 1.496)	1.366 (1.296 to 1.436)^**^
Log-CMI	0.033 (0.016 to 0.049)	0.007 (−0.022 to 0.037)	−0.121 (−0.183 to −0.059)^**^

Mean levels with 95% confidence intervals in parentheses of each variable in non-, occasional, and regular drinkers are shown. Age, hemoglobin Alc, and histories of smoking and regular exercise were adjusted. In addition, BMI was adjusted in analysis of LDL-C/HDL-C ratio and AIP. Asterisks denote significant differences from nondrinkers (^**^p < 0.01).

### Odds ratios of occasional and regular drinkers versus nondrinkers for high levels of each lipid-related index

Table 8 shows results of logistic regression analysis for the relationship of alcohol drinking with abnormally high levels of each lipid-related index. Both in univariate and multivariate analyses, odds ratios of regular drinkers versus nondrinkers for high LDL-C/HDL-C ratio, high AIP, high LAP, and high CMI were significantly lower than the reference level. In the multivariate analysis, odds ratio of occasional drinkers versus nondrinkers for high LDL-C/HDL-C ratio, high LAP, and high CMI were also significantly lower than the reference level, whereas the odds ratio of occasional drinkers versus nondrinkers for high AIP was not significantly different from the reference level.

**Table 8.** Relationships of Alcohol Drinking with Abnormalities of Lipid-Related Indices in Women with Hyperglycemia

High lipid-related index	Drinkers		
	Non	Occasional	Regular
High LDL-C/HDL-C (≥2.80)			
Crude	1.00	0.78 (0.54 to 1.13)	0.26 (0.08 to 0.83)^*^
Adjusted	1.00	0.65 (0.47 to 0.90)^**^	0.29 (0.09 to 0.94)^*^
High AIP (≥ −0.0649)			
Crude	1.00	0.88 (0.72 to 1.07)	0.61 (0.41 to 0.90)^*^
Adjusted	1.00	0.85 (0.69 to 1.06)	0.65 (0.43 to 0.99)^*^
High LAP (≥21.1)			
Crude	1.00	0.86 (0.69 to 1.07)	0.52 (0.36 to 0.77)^**^
Adjusted	1.00	0.79 (0.68 to 0.93)^**^	0.52 (0.35 to 0.78)^**^
High CMI (≥0.800)			
Crude	1.00	0.82 (0.67 to 1.01)	0.46 (0.32 to 0.68)^**^
Adjusted	1.00	0.73 (0.62 to 0.85)^**^	0.41 (0.28 to 0.62)^**^

Odds ratios with 95% confidence intervals in parentheses for each variable of occasional or regular drinkers versus nondrinkers are shown. In multivariate analysis, age, hemoglobin A1c, and histories of smoking and regular exercise were adjusted. In addition, BMI was adjusted in analysis of high LDL-C/HDL-C ratio and high AIP. Asterisks denote significant differences from the reference level of 1.00 (^*^p < 0.05; ^**^p < 0.01).

### Comparison of each component of the lipid-related indices in drinkers and nondrinkers

Table 9 shows mean levels of the four components of the lipid-related indices in non-, occasional, and regular drinkers. Both in univariate and multivariate analyses, waist-to-height ratio and LDL-C were significantly lower in regular drinkers than in nondrinkers, whereas log-transformed triglyceride level was not significantly different in regular drinkers and nondrinkers. HDL-C was significantly higher in occasional and regular drinkers than in nondrinkers.

**Table 9.** Comparison of Mean Levels of the Components of Lipid-Related Indices Among Non-, Occasional, and Regular Drinkers in Women with Hyperglycemia

Component of lipid-related index	Drinkers		
	Non	Occasional	Regular
Waist-to-height ratio			
Univariate	0.543 (0.540 to 0.547)	0.541 (0.535 to 0.548)	0.516 (0.504 to 0.528)^**^
Multivariate	0.543 (0.539 to 0.546)	0.542 (0.536 to 0.548)	0.519 (0.506 to 0.532)^**^
Log-triglycerides, mg/dL			
Univariate	2.053 (2.041 to 2.065)	2.048 (2.026 to 2.069)	2.011 (1.962 to 2.060)
Multivariate	2.052 (2.040 to 2.064)	2.047 (2.027 to 2.068)	2.030 (1.986 to 2.074)
HDL-C, mg/dL			
Univariate	58.3 (57.6 to 59.0)	60.5 (59.1 to 61.8)^*^	69.7 (66.5 to 72.9)^**^
Multivariate	58.2 (57.5 to 58.9)	60.9 (59.7 to 62.1)^**^	68.5 (65.9 to 71.0)^**^
LDL-C, mg/dL			
Univariate	130.8 (129.3 to 132.4)	128.2 (125.6 to 130.8)	121.0 (114.2 to 127.8)^**^
Multivariate	130.7 (129.1 to 132.2)	128.5 (125.8 to 131.2)	122.5 (116.7 to 128.2)^*^

Mean levels with 95% confidence intervals in parentheses of each variable in non-, occasional, and regular drinkers are shown. In multivariate analysis, age, hemoglobin A1c, and histories of smoking and regular exercise were adjusted. In addition, BMI was adjusted in analysis of variables, except for waist-to-height ratio. Asterisks denote significant differences from nondrinkers (^*^p < 0.05; ^**^p < 0.01).

## Discussion

This study for the first time showed the relationships of smoking and alcohol drinking with recently proposed lipid-related indices in women with hyperglycemia: Smoking was positively associated with high LDL-C/HDL-C ratio, AIP, and CMI, whereas alcohol drinking was inversely associated with high LDL-C/HDL-C ratio, AIP, LAP, and CMI. The lipid-related indices are determined by variables of blood lipids and adiposity. Smoking is known to be a risk factor of dyslipidemia,^17^ and that was also confirmed in this study: triglycerides and LDL-C were significantly higher in smokers than in nonsmokers, while HDL-C was significantly lower in smokers than in nonsmokers (Table 5). Therefore, the associations of smoking with LDL-C/HDL-C ratio, AIP, and CMI are explained by deteriorating effects of smoking on the lipid profile. On the other hand, there is a paradoxical relationship between smoking and obesity: although smokers have lower BMI than nonsmokers, smokers have a more metabolically adverse fat distribution profile with higher central adiposity.^19,20^ However, in the present study, waist-to-height ratio was significantly lower in smokers than in nonsmokers (Table 5). This dissociation might be related to the profile of the subjects with hyperglycemia, and mean WC was in fact relatively high in the subjects of this study (Table 1). Accordingly, the diverse relationships of smoking with high triglycerides and visceral obesity explain the results showing no significant difference in LAP, which is determined by using triglycerides and WC, in smokers and nonsmokers.

The risk of coronary artery disease in a general population is known to be lower in light-to-moderate alcohol drinkers than in nondrinkers.^36^ Light-to-moderate drinking has also been reported to be associated inversely with the risk of cardiovascular disease in patients with diabetes.^37,38^ The beneficial effect of alcohol on cardiovascular health is explained mainly by its action on lipid metabolism: HDL-C and LDL-C are higher and lower, respectively, in drinkers than in nondrinkers.^18^ These relationships were also found in this study using a database for women with hyperglycemia: HDL-C tended to be higher with an increase in the frequency of drinking and LDL-C was significantly lower in regular drinkers than in nondrinkers (Table 9). Habitual alcohol drinking has been reported to show a J-shaped relationship with triglyceride level.^39^ In this study, there was no significant difference in triglyceride levels of drinkers and nondrinkers (Table 9), which might be due to relatively low alcohol intake of the subjects of this study. The results of previous studies regarding whether alcohol drinking is a risk factor for obesity have been controversial.^40,41^ In this study, waist-to-height ratio was significantly lower in regular drinkers than in nondrinkers (Table 9), which agrees with the finding in a previous study that there was an inverse association between alcohol consumption and obesity in Japanese men.^42^ Therefore, the inverse associations of alcohol drinking with LDL-C/HDL-C ratio, AIP, LAP, and CMI found in this study are explained by the beneficial effects of alcohol on cholesterol metabolism and its suppressive effect on adiposity.

It has been shown that there is a strong association between proportions of smokers and drinkers^43^: smokers are more prone to drink than are nonsmokers and drinkers are more prone to smoke than are nondrinkers. However, the relations of smoking and drinking with lipid-related indices were not altered in multivariate analyses adjusting for drinking and smoking, respectively. Moreover, the relations of smoking and drinking with the lipid-related indices were found to be opposite in this study. Therefore, the associations of smoking and alcohol drinking with lipid-related indices are thought not to be confounded by alcohol drinking and smoking, respectively.

The results of this study suggest that LDL-C/HDL-C, AIP, and CMI are sensitive indices reflecting the influences of lifestyles such as smoking and alcohol drinking on cardiovascular risk. The amount of alcohol consumptions, the amount of smoking, and the proportions of drinkers and smokers are smaller in women than in men.^44–46^ In fact, the proportions of smokers and regular drinkers in women with hyperglycemia in this study were only 13.1% and 5.2%, respectively. Patients with diabetes are prone to have a high risk of cardiovascular disease,^1^ which is known to be partly explained by the high incidence of dyslipidemia.^3,4^ The results of this study for women with hyperglycemia showed that even a small amount of smoking aggravates dyslipidemia and increases the risk of cardiovascular disease, whereas habitual alcohol drinking lowers the risk if not heavy drinking. In a general population, the adjusted odds ratio for high CMI in female smokers versus nonsmokers was reported to be 1.41 (1.30 to 1.53),^23^ which is similar to the odds ratio in women with hyperglycemia in this study (Table 4). In a general population, the odds ratio for high CMI in male light smokers versus nonsmokers was 1.16 (1.10 to 1.23).^24^ Since 99.4% of the smokers were light smokers (less than 21 cigarettes per day) in the present study, and men tend to smoke more than women, the association of smoking with high CMI is thought to be stronger in women than in men. This might be related to higher cardiovascular risk in women with diabetes than in men with diabetes.^29^

There are limitations of this study. The proportions of heavy smokers (21 cigarettes or more per day) and heavy drinkers (22 g ethanol per day or more) were very low (only 0.60% [*n* = 13] and 2.9% [*n* = 63], respectively), and the population size was not large enough to evaluate amounts of smoking and alcohol intake in relation to lipid-related indices in this study. In addition, the number of participants with diabetes (*n* = 421) was not large enough to investigate the relationships of smoking and alcohol drinking with lipid-related indices separately in patients with diabetes. Thus, further studies are needed to determine the relationships between heavy drinking and lipid-related indices since there is a J-shaped relationship between alcohol drinking and triglyceride level.^39^ In the multivariate analyses in this study, age, history of regular exercise, BMI, and hemoglobin A1c were used as covariates and explanatory variables. However, there are other possible confounders, for example, diet, socioeconomic factors, and hormonal status, for the relations of smoking and alcohol drinking with lipid-related indices. Finally, since this study is cross-sectional in its design, further prospective studies and intervention trials are needed to discuss causal relationships between lifestyles and lipid-related indices.

## Conclusions

In women with hyperglycemia, smoking was positively associated with LDL-C/HDL-C ratio, AIP, and CMI, and habitual alcohol drinking was inversely associated with LDL-C/HDL-C ratio, LAP, AIP, and CMI. There were inverse associations of smoking and alcohol drinking with adiposity, whereas the relations of smoking and alcohol drinking with lipids were diverse: HDL-C was lower in smokers than in nonsmokers and was higher in drinkers than in nondrinkers, and LDL-C was higher in smokers than in nonsmokers and was lower in drinkers than in nondrinkers. Thus, lipid-related indices reflect different effects of smoking and alcohol drinking on blood lipids. LDL-C/HDL-C ratio, AIP, and CMI are thought to be affected by both smoking and alcohol drinking, which accelerates and suppresses atherosclerotic progression, respectively.

## Abbreviations Used

AIP: atherogenic index of plasma
ANCOVA: analysis of covariance
ANOVA: analysis of variance
BMI: body mass index
CMI: cardiometabolic index
HDL-C: high-density lipoprotein cholesterol
JDS: Japan Diabetes Society
LAP: lipid accumulation product
LDL-C: low-density lipoprotein cholesterol
NGSP: National Glycohemoglobin Standardization Program
TG: triglyceride
WC: waist circumference
